# Pharmacoinformatics and Preclinical Studies of NSC765690 and NSC765599, Potential STAT3/CDK2/4/6 Inhibitors with Antitumor Activities against NCI60 Human Tumor Cell Lines

**DOI:** 10.3390/biomedicines9010092

**Published:** 2021-01-19

**Authors:** Bashir Lawal, Yen-Lin Liu, Ntlotlang Mokgautsi, Harshita Khedkar, Maryam Rachmawati Sumitra, Alexander T. H. Wu, Hsu-Shan Huang

**Affiliations:** 1 PhD Program for Cancer Molecular Biology and Drug Discovery, College of Medical Science and Technology, Taipei Medical University and Academia Sinica, Taipei 11031, Taiwan; d621108004@tmu.edu.tw (B.L.); d621108006@tmu.edu.tw (N.M.); d621108005@tmu.edu.tw (H.K.); maryamrachma60@gmail.com (M.R.S.); 2 Graduate Institute for Cancer Biology and Drug Discovery, College of Medical Science and Technology, Taipei Medical University, Taipei 11031, Taiwan; 3 Department of Pediatrics, Taipei Medical University Hospital, Taipei 11031, Taiwan; yll.always@gmail.com; 4 Taipei Cancer Center, Taipei Medical University, Taipei 11031, Taiwan; 5 Department of Medicine, School of Medicine, College of Medicine, Taipei Medical University, Taipei 11031, Taiwan; 6 TMU Research Center of Cancer Translational Medicine, Taipei Medical University, Taipei 11031, Taiwan; 7 The PhD Program of Translational Medicine, College of Medical Science and Technology, Taipei Medical University, Taipei 11031, Taiwan; 8 Clinical Research Center, Taipei Medical University Hospital, Taipei Medical University, Taipei 11031, Taiwan; 9 Graduate Institute of Medical Sciences, National Defense Medical Center, Taipei 11490, Taiwan; 10 School of Pharmacy, National Defense Medical Center, Taipei 11490, Taiwan; 11 PhD Program in Biotechnology Research and Development, College of Pharmacy, Taipei Medical University, Taipei 11031, Taiwan

**Keywords:** protein-ligand interaction, molecular docking simulation, target identification, small-molecule derivatives of salicylanilide, drug discovery, drug development

## Abstract

Signal transducer and activator of transcription 3 (STAT3) is a transcriptional regulator of a number of biological processes including cell differentiation, proliferation, survival, and angiogenesis, while cyclin-dependent kinases (CDKs) are a critical regulator of cell cycle progression. These proteins appear to play central roles in angiogenesis and cell survival and are widely implicated in tumor progression. In this study, we used the well-characterized US National Cancer Institute 60 (NCI60) human tumor cell lines to screen the in vitro anti-cancer activities of our novel small molecule derivatives (NSC765690 and NSC765599) of salicylanilide. Furthermore, we used the DTP-COMPARE algorithm and in silico drug target prediction to identify the potential molecular targets, and finally, we used molecular docking to assess the interaction between the compounds and prominent potential targets. We found that NSC765690 and NSC765599 exhibited an anti-proliferative effect against the 60 panels of NCI human cancer cell lines, and dose-dependent cytotoxic preference for NSCLC, melanoma, renal, and breast cancer cell lines. Protein–ligand interactions studies revealed that NSC765690 and NSC765599 were favored ligands for STAT3/CDK2/4/6. Moreover, cyclization of the salicylanilide core scaffold of NSC765690 mediated its higher anti-cancer activities and had greater potential to interact with STAT3/CDK2/4/6 than did NSC765599 with an open-ring structure. NSC765690 and NSC765599 met the required safety and criteria of a good drug candidate, and are thus worthy of further in-vitro and in-vivo investigations in tumor-bearing mice to assess their full therapeutic efficacy.

## 1. Introduction

Despite advances in biomedical research, cancer remains a public health concern and is currently ranked the second leading cause of global mortality [1,2]. The etiology of cancer is often multifactorial, involving an interplay between genetic and epigenetic factors which amount to dysregulation of molecular networks, proteins, RNA, and DNA in favor of cell growth and proliferation [3,4]. The survival, growth, and metastasis of tumor cells depend on cellular differentiation, proliferation, angiogenic, and apoptotic mechanisms [5], which are controlled by a range of protein kinases and signal transduction pathways [6,7]. Cyclin-dependent kinases (CDKs) are a family of serine/threonine protein kinases with catalytic and regulatory subunits [8]. Out of the nine CDKs so far identified, four (CDK1, CDK2, CDK4, and CDK6) play important regulatory roles in the cell cycle; CDK4/D-cyclin, CDK2/E-cyclin, and CDK6/D-cyclin regulate the G_1_ to S phase of the cell cycle transition, while the complex of CDK1 or CDK2 with cyclin A regulates the S to G_2_ transition [9]. Under physiological conditions, the catalytic and regulatory (cyclin) subunits of CDKs remain dissociated. However, periodic complexation of a cyclin with its catalytic unit leads to its activation and phosphorylation of a variety of downstream target proteins required for cell cycle progression [10,11]. CDKs regulate cell cycle transitions via phosphorylation and subsequent inactivation of the retinoblastoma (Rb) protein, a tumor suppressor that prevents cell cycle transition [12]. Thus, the inactivation of this Rb protein allows the free flow and progression of cells into the cell cycle, leading to multi-cell cycles, cell proliferation, and eventual development into cancer cells [12].

A number of key oncogenic abnormalities, including amplification of cyclin D; inactivation of CDKN2A (p16); and deletions/mutations upstream of cyclin D, such as activating mutations of phosphatidylinositol 4,5-bisphosphate 3-kinase catalytic subunit alpha (PIK3CA)/the B-raf proto-oncogene, serine/threonine kinase (BRAF), and phosphatase and tensin homolog (PTEN) deletion [13,14], were identified as contributing factors to hyperactive CDKs and consequently deregulated the cell cycle. Therefore, since dysfunctional cell cycle regulation via oncogenic aberrations of CDKs is a hallmark of all human cancers [15], pharmacological targeting of CDKs will undoubtedly affect cancer proliferation and survival [16,17]. Hence, CDK inhibitors have been developed and evaluated; however, disappointingly, while first-generation inhibitors of CDK were non-selective and besieged with toxicity [18,19], second-generation CDK4/6 inhibitors, although showing promising outcomes, are plagued with acquired resistance, which develops in almost all cases [20] due to the activation of other oncogenic pathways, including c-Myc, signal transducer and activator of transcription 3 (STAT3), and phosphatidylinositol 3-kinase (PI3K)/AKT/mammalian target of rapamycin (mTOR) signaling pathways [21,22].

STAT3 is a cytoplasmic transcription factor involved in a number of biological processes including cell differentiation, proliferation, survival, and angiogenesis [23]. Overexpression of STAT3 is associated with poor clinical prognoses of cancer patients [24]. It is therefore not surprising that the STAT3 signaling axis has long been explored in cancer therapy owing to its roles in tumor formation, metastasis, and therapeutic failure [25,26]. Preclinical studies revealed that aberrant STAT3 expression mediates immunosuppression of tumor cells [27,28], while inhibition of STAT3 re-sensitizes therapy-resistance breast cancer cells to palbociclib treatment [29]. Summing up the above clinical and preclinical evidence, it is convincing that identifying and validating novel CDK inhibitors capable of simultaneously targeting STAT3 signaling may open up new windows for long-lasting and multilayered tumor control [30].

The translational value of knowledge of cancer biology into developing effective prognostic/diagnostic and therapeutic strategies for clinical practice remains disappointing [31]. However, increasing knowledge of the molecular basis of tumorigenesis, applications of multi-omics approaches, and molecular simulations based on a structural analysis of receptor–ligand interactions have jointly contributed to the identification of more-reliable predictive markers and the discovery of novel, less-toxic, and target-specific anticancer agents [31,32,33,34]. At present, substantial attention is being focused on small molecules for targeted therapy in cancer treatments [35].

NSC765599 and NSC765690 are small molecule derivatives of salicylanilides (PubChem CID: 60202556; NDMC101), which were previously synthesized and evaluated for biological activities in our Lab [36]. We had previously conducted a series of chemical modifications of the lead molecule, NDMC101, to yield the open ring (NSC765599) [37] and close ring (NSC765690) [38] derivatives. Herein we demonstrated that both NSC765690 and NSC765599 exerted antitumor activities in vitro against panels of NCI60 human tumor cell lines. We further identified and validated CDK2/4/6 and STAT3 as druggable candidates for the compounds, through in silico and molecular simulation of ligand–receptor interaction studies. Hence, our data provide evidence that expressions of CDK2/4/6 and STAT3 can be directly regulated by NSC765690 and NSC765599 with consequent antitumor implications in multiple cancer types.

## 2. Materials and Methods

### 2.1. In Vitro Anticancer Screening against 60 Full NCI Cell Panels of Human Tumor Cell Lines

**Figure 1:**
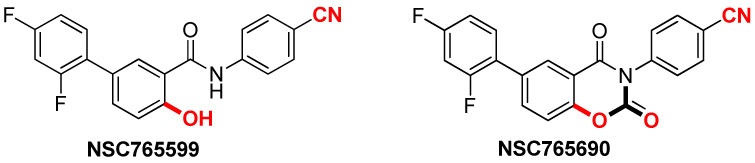
Chemical structures of NSC765599 (N-(4-cyanophenyl)-2′,4′-difluoro-4-hydroxy-[1,10-biphenyl]-3-carboxamide) and NSC765690 (4-(6-(2,4-Difluorophenyl)-2,4-dioxo-2H-benzo[e][1,3]-oxazin-3(4H)-yl) benzonitrile).

NSC765690 and NSC765599 (Figure 1) were submitted to the National Cancer Institute (NCI) for the screening of its panel of NCI60 cancer cell lines. The preliminary single-dose screening of the two compounds was conducted against 60 full NCI cell line panels comprising melanomas, leukemia, central nervous system (CNS) cancers, NSCLC, renal cancer, breast cancer, ovarian cancer, and prostate cancer in agreement with the protocol of the NCI. Following single-dose testing at 10 μM, the two compounds were selected for five-dose screening against the same panels of cancer cell lines. As described previously [39,40], protocols for NCI60 cell five-dose screening involved seeding of about 5000~40,000 cells/well (depending on the doubling time of individual cell lines) in 96-well plates, followed by treatment with NSC765690 or NSC765599 at concentrations of 0.01, 0.1, 1.0, 10, and 100 μM and incubation at 37 °C in 5% humidified CO_2_ for 48 h. Cells were fixed with a sulforhodamine B (SRB) solution [41] followed by a series of washing and staining to determine their viability. Growth inhibition was calculated relative to cells without drug treatment and time-zero control. Results of the five-dose assay are represented in terms of a dose-dependent curve, tumor growth inhibition (TGI), LC_50_ (concentration needed to kill 50% of cells by cytotoxic activity) for each cell line tested, and GI_50_ (concentration needed to inhibit 50% of cancer cell growth) [42]. Results were presented as cell growth relative to the untreated cell control and to the time zero number of cells. Growth inhibitions were indicated by values between 0 and 100, while lethality (cytotoxic effect) was indicated by values less than 0.

### 2.2. Identifying the Molecular Targets and Therapeutic Classes of NSC765599 and NSC765690

NSC765599 and NSC765690 were screened for potential drug target using SwissTarget prediction algorithm, which predicts potential drug target based on the principle of similarity [43]. In addition, we also employ the computer-aided Prediction of Biological Activity Spectra (PASS) web resources to predict the potential drug targets [44]. The activity patterns (fingerprints) of both NSC765599 and NSC765690 were correlated to NCI synthetic compounds, standard agents and molecular targets using the DTP-COMPARE algorithms [45]. The NSC numerical IDs were used as “seed” while GI_50_, TGI and LC_50_ were set as the endpoints.

### 2.3. In Silico Molecular Docking Analyses

The three-dimensional (3D) structure of palbociclib (CID: 5330286) and SH-4-54 (CID: 72188643) were retrieved in SDF file format from the PubChem database, while the 3D structures of NSC765690 and NSC765599 were drawn out in sybyl mol2 format using the Avogadro molecular builder and visualization tool vers. 1.XX [46] and were subsequently transformed into the protein data bank (PDB) format using the PyMOL Molecular Graphics System, vers. 1.2r3pre (Schrödinger, LLC). The PDB file of the 3D structure of the receptors and crystal structures of apo CDK2 (PDB; 4EK3), CDK4/cyclin D3 (PDB; 3G33), CDK6/cyclin (PDB; 1JOW), and STAT3 (PDB; 4ZIA), were retrieved from the Protein Data Bank. The PDB file formats of the ligands (NSC765690, NSC765599, and palbociclib) and the receptors (STAT3; CDK2, 4, and 6) were subsequently converted into the Auto Dock Pdbqt format using AutoDock Vina (vers. 0.8, The Scripps Research Institute, La Jolla, CA, USA) [47]. Pre-docking preparation of the receptors followed the removal of water molecules, while hydrogen atoms and Kolmman charges were added accordingly. Molecular docking studies were performed using Autodock VINA software and by following the protocols described in our previous study [3]. The docking results based on hydrogen bonds and electrostatic and hydrophobic interactions of the best pose of the ligand–receptor complexes were expressed as binding energy values (kcal/mol). PyMOL software was used to visualize H-bond interactions, binding affinities, interacting amino acid residues, binding atoms on the ligands and receptors, and 3D graphical representations of ligand-receptor complexes, while 2D graphical illustrations of ligand-binding interactions were further visualized using Discovery studio visualizer vers. 19.1.0.18287 (BIOVIA, San Diego, CA, USA) [48].

### 2.4. Pharmacokinetics, Drug-Likeness, Toxicity and Medicinal Chemical Analyses

The drug-likeness, medicinal chemistry, and pharmacokinetics, including the adsorption, distribution, metabolism, excretion, and toxicity (ADMET) properties of NSC765690 and NSC765599 were analyzed using SwissADME software developed by the Swiss Institute of Bioinformatics [49]. The drug-likeness properties were analyzed in terms of Ghose (Amgen), Egan (Pharmacia), and Veber (GSK), and more importantly, the Lipinski (Pfizer) rule-of-five, as well as cLogP, molecular mass, hydrogen acceptor, hydrogen donor, and molar refractive index [50] for drug-likeness and drug discovery. The Abbot Bioavailability Score was calculated based on the probability of the compound to have at least 0.1 (10%) oral bioavailability in rats or measurable Caco-2 permeability [51], while gastrointestinal absorption and brain penetration properties were analyzed using the Brain Or IntestinaL EstimateD permeation (BOILED-Egg) model [52]. The acute toxicity (LD_50_) in rats and environmental toxicity were predicted using GUSAR software [53].

### 2.5. Data Analysis

Spearman’s rank correlation was used to assess the correlations of NSC765599 and NSC765690 fingerprints with NCI synthetic compounds, standard agents, and molecular targets. The COMPARE correlation threshold was set to ≥45 common cell lines, ≥0.1 correlation coefficient, and ≥0.05 standard deviation. The growth inhibition by NSC765690 and NSC765599 in single-dose assay was obtained by subtracting the positive value on the plot from 100, i.e., a value of 40 would mean 60% growth inhibition.

## 3. Results

### 3.1. NSC765690 and NSC765599 Exhibited Anti-Proliferative Effects on NCI60 Human Cancer Cell Lines

**Figure 2:**
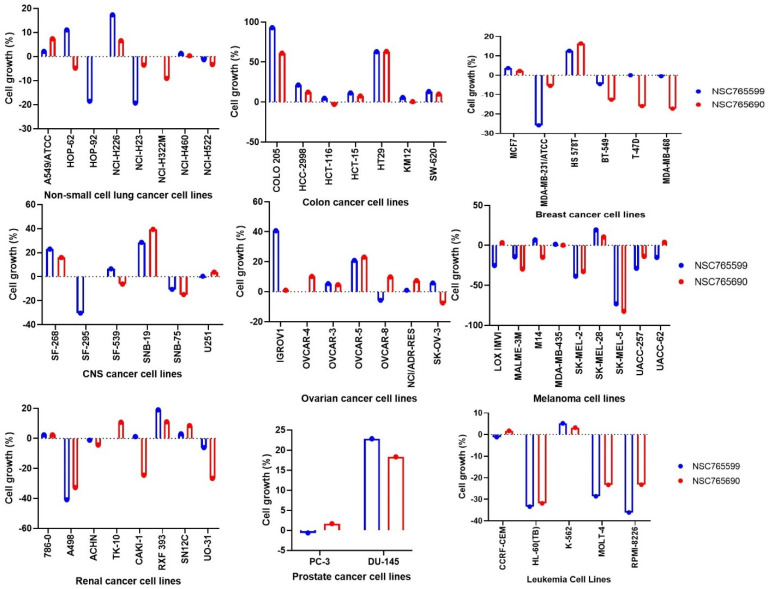
Anti-proliferative effect of NSC765690 and NSC765599 against panels of 60 human cancer cell lines. Each cell line was treated with a single dose of (10 μM) of each compound. The zero points denote the mean percentage of cell growth. The percentage growth inhibition of each cell line relative to the mean is represented by values under 100, whereas those values below 0 indicate cell death.

Both NSC765690 and NSC765599 exhibited anti-proliferative effect against all the 60 panels of NCI human cancer cell lines (Figure 2). Furthermore, single-dose treatment with NSC765690 and NSC765599 also demonstrated cytotoxic effects against some cell lines. As indicated by the percentage growth altered by treatment, melanoma (SK-MEL-5, SK-MEL-2, and MALME-3M), renal (A498, UO-31, and CAKI-1), leukemia (HL-60, molt-4, and RPMI-8226), and breast (MDA-MB-468, T-47D, and HS 578T) cancer cell lines were the most responsive to NSC765690 treatment. For NSC765599, melanoma, renal cancer, leukemia, and the breast cancer cell line were also sensitive to cytotoxic effects of NSC765599 at 10 μM. However, panels of prostate, ovarian, and colon cancer cell lines were less responsive to NSC765690 and NSC765599 treatment (Figure 2). These primary single dose screening results clearly indicated the anti-proliferative activities of NSC765690 and NSC765599 against different kinds of human cancer cell lines, and thus are worthy of further evaluation for dose-dependent activities.

### 3.2. NSC765690 and NSC765599 Exhibited Dose-Dependent Cytotoxic Effects against NCI 60 Human Cancer Cell Lines

**Figure 3:**
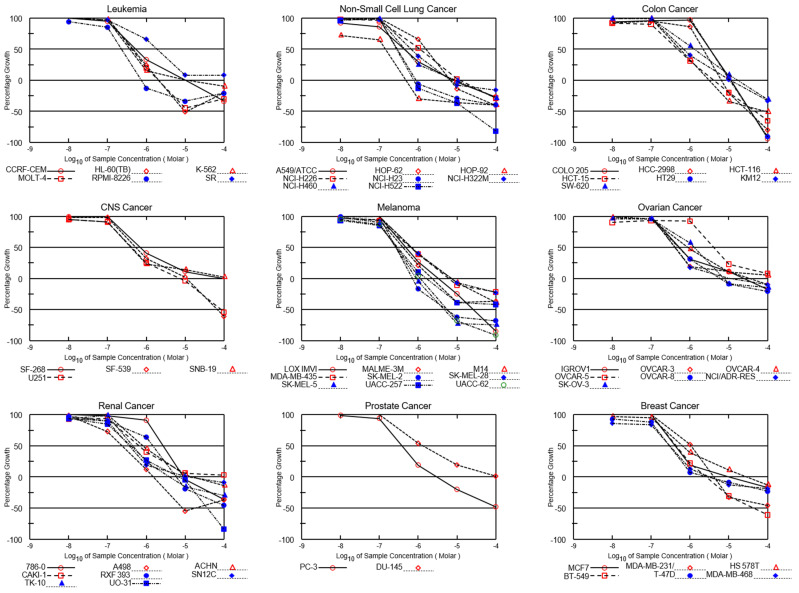
Dose–cytotoxic response curves of NSC765690 against panels of 60 NCI human cancer cell lines. The growth percentage value of +100 on the *Y*-axis represents the growth of untreated cells, the 0 value represents no net growth, while −100 represents the complete death of cells.

**Figure 4:**
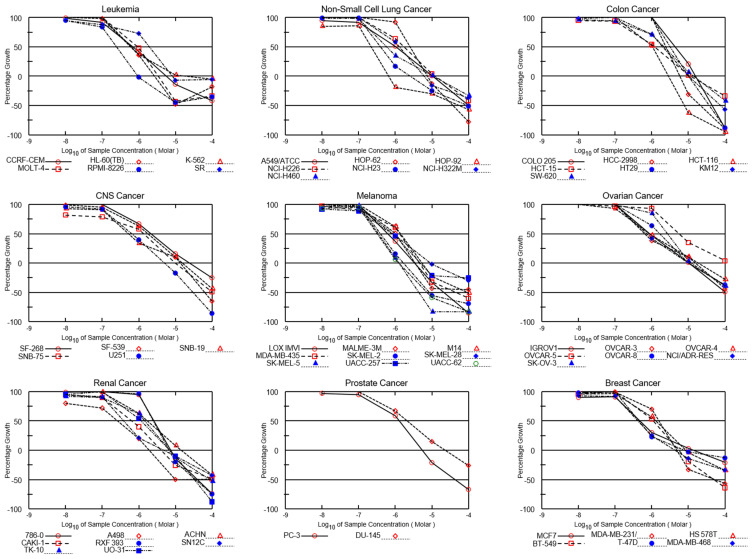
Dose–cytotoxic response curves of NSC765599 against panels of 60 NCI human cancer cell lines. The growth percentage value of +100 on the *Y*-axis represents the growth of untreated cells, the 0 value represents no net growth, while −100 represents the complete death of cells.

Both NSC765690 and NSC765599 exhibited dose-dependent cytotoxic activities against melanoma (SK-MEL-5, SK-MEL-2, and MALME-3M), NSCLC (HOP-92, A549/ATCC, NCI-H23, NCI-H522, and NCI-H522), CNS (SF-268, SNB-19, and U251), renal (A498, UO-31, and CAKI-1), leukemia (HL-60, molt-4, and RPMI-8226), and breast (MDA-MB-468, T-47D, and HS 578T) cancer cell lines (Figure 3 and Figure 4). The GI_50_ values ranged from 0.14~2.79 μM in NSC76569-treated cell lines and 0.219~5.55 μM in NSC765599-treated cell lines. Consistent with the activities demonstrated in single-dose treatments, the NSCLC, melanoma, renal, and breast cancer cell lines were most sensitive to NSC765690 treatment, while leukemia, CNS, ovarian, and colon cancer cell lines were the least sensitive to NSC765690 (Table 1). Analysis of LC_50_ values also indicated that the melanoma cell lines were the most sensitive to drug treatments, with NSC765690 being the most active (Table 1).

### 3.3. NSC765599 and NSC765690 Shared Similar NCI Anti-Cancer Fingerprints and Molecular Targets of Cell Cycle Transition Proteins

DTP-COMPARE analysis results showed that the 10 NCI synthetic compounds that are most correlated with NSC765690 and NSC765599 anti-cancer fingerprints are small molecules (MW: 168.19~506.5 g/mol), with *p* values in the range of 0.44~0.62. In addition, NSC765690 (*p*-value range of 0.33~0.74) and NSC765599 (*p*-value range of 0.15~0.36) shared anticancer mechanism with a number of standardized drugs in the NCI database (Table 2). The molecular target fingerprints generated also showed that both compounds shared a similar positive correlation (*p* = 0.1~0.3) with the expression of multiple genes, most of which are proteins involved in cell cycle progressions (Table 3).

### 3.4. CDK2/4/6 and STAT3 Are Potential Druggable Candidates for NSC765690 and NSC765599

Using NSC765690 and NSC765599 as query molecules on the SwissTargetPrediction algorithm, a computer-based drug target prediction tool that identifies the most probable macromolecular targets of a small molecule, on the basis of similarity with a known actives compound in the library [54], we identified a number of targetable proteins, most of which are CDKs and associated proteins. Among these predicted targets, we found that kinases, cytochrome P450, enzymes, and electrochemical transporters were the most occurring targeted classes (Table 4, Supplementary Figure S1). Specifically, STAT3, four members of CDKs (CDK1/2/4/9), and three cyclins were among the top-ranked targeted proteins, while NSC765599 was predicted to target CDK2/4/5/6. Other top-ranked proteins targetable by NSC765599 are shown in Table 4. Coherent with the SwissTarget prediction, the PASS analysis of NSC765690 and NSC765699 also predicted (all pa > pi) inhibitions of CDKs and STAT3 amongst other activities (Table 5).

### 3.5. Molecular Docking Reavealed Favoured Ligandability of NSC765690 and NSC765599 for CDK2/4/6 and STAT3

**Figure 5:**
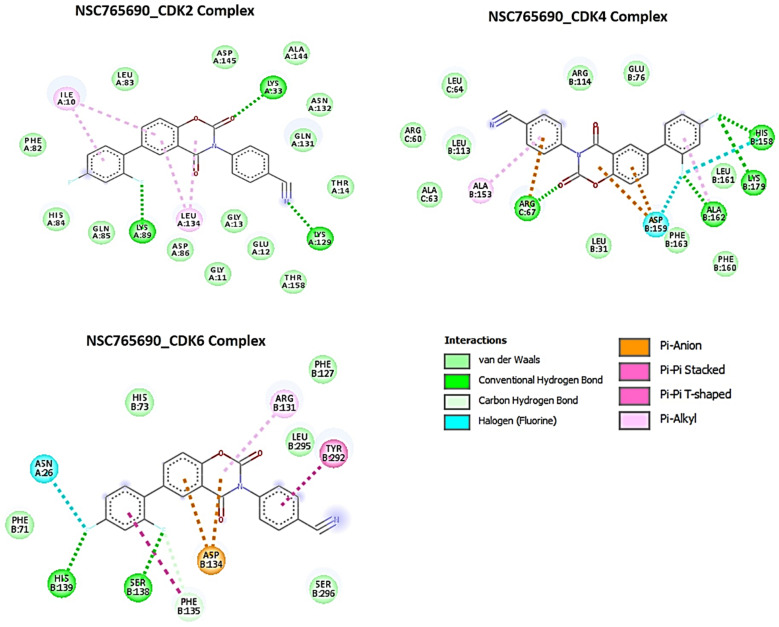
Docking profiles of NSC765690 with CDK2, CDK4, and CDK6. Two dimensional (2D) representations of ligand–receptor interactions occurring between NSC765690 and the target receptors (CDK2/4/6). The deep-green color indicates the strongest ligand–receptor interaction (due to conventional hydrogen bonds) of the best docking pose.

**Figure 6:**
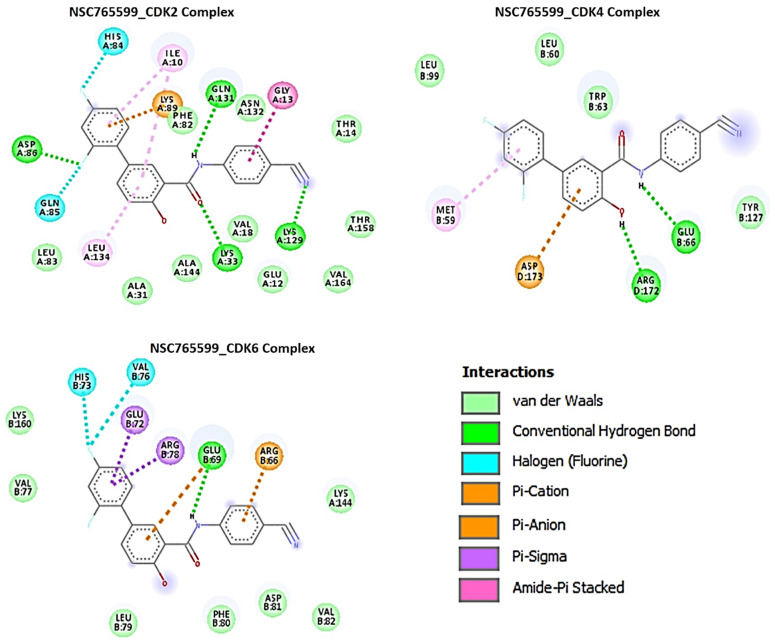
Docking profiles of NSC765599 with CDK2, CDK4, and CDK6. Two dimensional (2D) representations of ligand–receptor interactions occurring between NSC765599 and the target receptors (CDK2/4/6). The deep-green color indicates the strongest ligand–receptor interaction (due to conventional hydrogen bonds) of the best docking pose.

**Figure 7:**
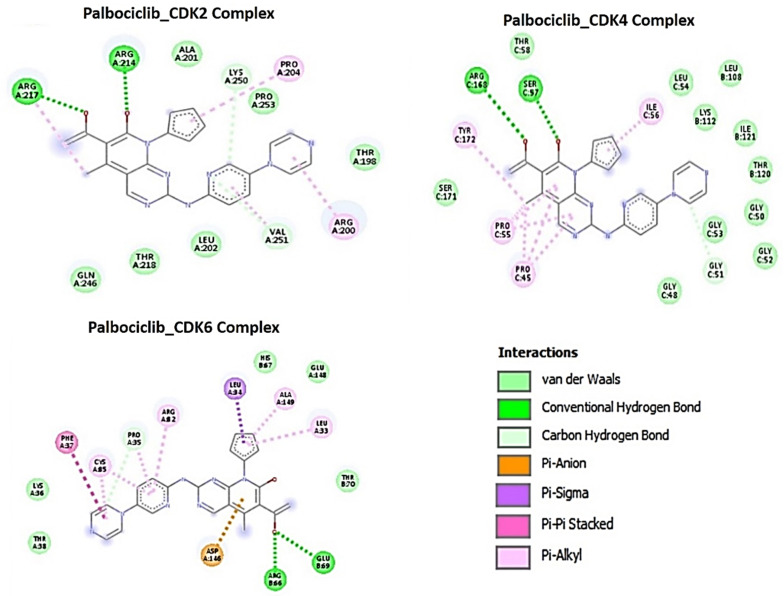
Docking profiles of palbociclib with CDK2, CDK4, and CDK6. Two dimensional (2D) representations of ligand–receptor interactions occurring between palbociclib and the target receptors (CDK2/4/6). The deep-green color indicates the strongest ligand–receptor interaction (due to conventional hydrogen bonds) of the best docking pose.

Using molecular docking studies, we found that both NSC765690 and NSC765599 exhibited strong interactions with the crystal structure of apo CDK2 (PDB; 4EK3), CDK4/cyclin D3 (PDB; 3G33), CDK6/cyclin (PDB; 1JOW), and STAT3 (PDB; 4ZIA). The observed interactions and binding affinities indicated that CDK2 is the most favored receptor for both compounds (Figure 5 and Figure 6, Table 6, Supplementary Figures S2–S4), while NSC765690 exhibited stronger interactions with CDK2/4/6 than does NSC765599. Furthermore, we compared the docking profiles of the two compounds with a standard CDK inhibitor, palbociclib (Figure 7), and found that NSC765690 demonstrated stronger binding affinities with CDK2/4/6 compared to NSC765599 and palbociclib. Docking of all ligands (NSC765690, NSC765599, and palbociclib) exhibited short binding distances with the receptors CDK2 (2.14~3.36 Å), CDK4 (1.87~3.23 Å), and CDK6 (2.31~3.17 Å) (Table 6).

**Figure 8:**
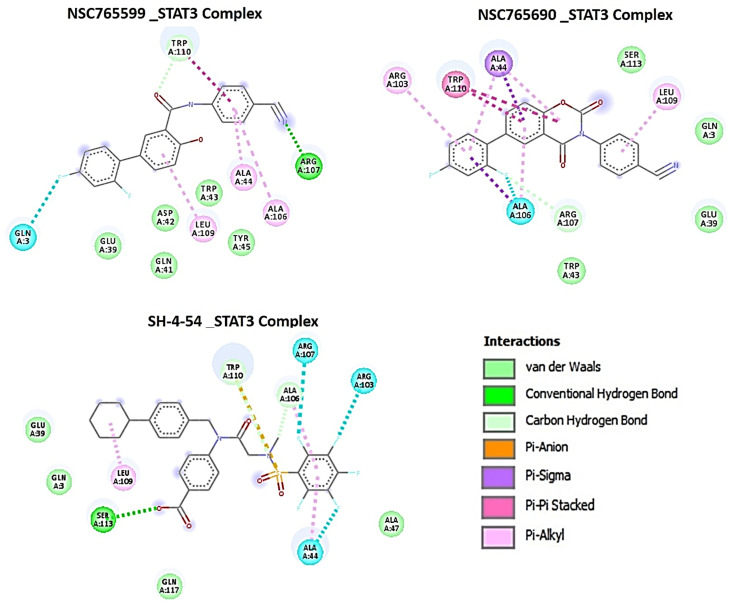
Comparative docking profiles of STAT3 with NSC765599, NSC765690, and SH-4-54 (a known STAT3 inhibitor). A 2D representation of ligand–receptor interactions of STAT3 with NSC765599, NSC765690, and SH-4-54 in the receptor binding pocket.

Our careful analysis of the interactions between NSC765690 and the receptors revealed a higher number of conventional H-bonding, pi interactions, and Van der Waal forces created on the ligand backbone with a higher number of amino acids residues than does NSC765599 and palbociclib, in the CDK2/4/6 binding cavities (Table 6). NSC765690-STAT3 and NSC765599-STAT3 complexes were stabilized by similar interactions. However, the overall binding affinity of STAT3 with a known inhibitor, SH-4-54, was less negative than the values observed for NSC765690-STAT3 and NSC765599-STAT3 complexes (Figure 8, Supplementary Figure S5).

### 3.6. NSC765599 and NSC765690 Met the Required Criteria of Drug-Likeness and Safety

NSC765690 met the required criteria of a good drug candidate in terms of lipophilicity, polarity, flexibility, solubility, saturation, and molecular weight, while NSC765599 slightly violated the required range of solubility (Log S(ESOL) = 0~6) and lipophilicity (XlogP3 = −0.7~5) having slight outlying values of −5.71 and 5.47 respectively. Both compounds demonstrated good synthetic accessibilities, highly probable GIA absorption, and bioavailability, but poor BBB permeation (Supplementary Figures S6 and S7, Table 7). Predicted ecotoxicity and acute toxicity for different administration routes of NSC765690 and NSC765599 produce class 4 and 5 levels of acute toxicity (LD_50_) according to OECD classification (Table 7). Collectively, NSC765599 and NSC765690 met the criteria for a drug-likeness candidate and are relatively known to be toxic.

## 4. Discussion

Despite advances in treatment modalities, cancer survival ratios are still disappointing; thus, developing a novel chemotherapeutic strategy may improve the prognosis of the cancer patient [55]. At present, substantial attention is being focused on small molecules for targeted therapy in cancer treatments [35]. In the present study, we demonstrated the anti-proliferative and dose-dependent cytotoxic effect of NSC765690 and NSC765599 against panels of NCI human cancer cell lines, with NSC765690 demonstrating higher activities than NSC765599. However, there was the least amount of cell lethality in panels of leukemia, CNS, ovarian, and colon cancer cell lines, suggesting that NSC765690 and NSC765599 are not generally toxic to growing cell lines, but display some degree of cytotoxic preference for NSCLC, melanoma, renal, and breast cancer cell line.

The NCI60 cell lines have been well-characterized for genetic and protein expression patterns, and, a computational tool, the DTP-COMPARE algorithm, has been developed to identify the molecular targets and mechanism(s) of action for the unknown compound from known drugs as well as the known molecular targets in the NCI databases [56]. Interestingly, our analysis of correlation patterns of NSC765690 anticancer fingerprint with mechanistically known NCI-Standard Agents suggests that NSC765690 might be targeting DNA replication and cell cycle. NSC765599, on the other hand, shares similar (*p* = 0.15~0.36) antitumor mechanistic fingerprints with known inhibitors of growth factor receptor and cell cycle transition, and DNA damage inducer. However, the modest correlation with NCI-standard agents suggests that NSC765599 may have a unique mechanism of action not common to standard agent database. The strong antitumor pattern correlation (*p* = 0.44~0.6) between NSC765690 and NSC765599 fingerprint with NCI synthetic compounds suggests a mechanism of action similar to that of the seed compounds. Unfortunately, the top-ranked correlated compounds antineoplastic-643812 (*p* = 0.61) and N-(3-chloro-2-methylphenyl)-2-hydroxy-3-nitrobenzamide (*p* = 0.62) are yet to be mechanistically investigated, while combretastatin A-4 with *p*-value 0.58 (NSC765690) and *p*-value 0.53 (NSC765599) have been reported for the mechanism of action, indicating cell cycle arrest and inhibition of tubulin [57], hence providing hypothesis about the possible mechanism of actions of NSC765690 and NSC765599. The high number of cell cycle proteins and cyclin and cyclin-dependent kinases identified as molecular target fingerprints of both NSC765690 and NSC765599 strongly suggest that cyclin and cyclin-dependent kinase pathways are the core molecular targets of NSC765690 and NSC765599 predicted by COMPARE. However, the correlations (*p* = 0.1~0.3) were low, indicating the possibility of a new mechanism not captured by COMPARE.

The COMPARE prediction, together with in-silico SwissTarget and PASS prediction collectively identified STAT3/CDK2/4/6 as the most probable target for NSC765690 and NSC765599, and thus were further studied for ligand–receptor interactions. Interestingly, we found that NSC765690 and NSC765599 docked well into the receptor cavities of STAT3, CDK2, CDK4, and CDK6, with CDK2 being the most favored receptor while CDK4 appeared to be the least favored. Comparatively, the higher binding affinities and closer proximity of NSC765690 to the receptors suggested that NSC765690 is a better ligand for CDK2, CDK4, and CDK6 than is NSC765599.

NSC765690 and NSC765599 are both derivatives of NDMC101. A close look at the structure-related activities of NSC765690 and NSC765599 revealed that cyclization of salicylanilide core scaffold (Figure 1) was the structural feature associated with enhanced anticancer activity and better docking profiles of NSC765690 with STAT3/CDK2/4/6 compared to NSC765599 with an open-ring structure (Figure 7 and Figure 8). Interestingly, when the same receptors (CDK2/4/6) were docked with palbociclib, a clinical CDK inhibitor, higher binding energies (lower binding affinities) were recorded for palbociclib compared to NSC765690, thus making the latter the more-favored ligand for CDK2/4/6.

The unique stability of NSC765690 in the binding sites of CDK2, CDK4, and CDK6 could be attributed to the larger number of H-bond and pi interactions including pi–cation, pi–anion, pi–alkyl, pi–pi stack, and pi–pi T-shaped interactions between NSC765690 and the receptors. The large numbers of pi interactions, which mostly involve charge transfer, help in intercalating the NSC765690 in the receptors’ binding cavities. The higher affinity of NSC765690 was also associated with the presence of a larger number of Van der Waal forces created on its backbone with respective amino acids Phe82, Leu83, Asp145, Ala144, Asn132, Gln131, Thr14, Thr158, Glu12, Gly13, Gly11, Asp86, Gln85 and His84 of CDK2; Leu64, Arg60, Leu113, Ala63, Leu31, Phe163, Phe160, Leu161, Glu76 and Arg114 of CDK4; and Phe71, Ser296, Leu295, Phe127, and His73 of CDK6, which undoubtedly created a strong cohesive environment, thereby stabilizing the complex formed [58]. These higher numbers of interactions undoubtedly contributed to the higher affinity that NSC765690 has for CDK2, CDK4, and CDK6 than do NSC765599 and palbociclib. Palbociclib has been actively applied in multiple preclinical models and was approved for targeting CDK4/6 as anticancer therapy for breast cancer; however, acquired resistance occurs in almost all cases [20]. Although docking STAT3 with a known inhibitor, SH-4-54, revealed a higher number of conventional H-bond and halogen bond interactions with STAT3, the pi interactions were fewer, and consequently, the overall binding affinity was less negative. Therefore, higher pi interactions also contributed to the higher binding affinity that NSC765690 has for STAT3 than does SH-4-54. Our molecular simulations of ligand–receptor interactions validated the computational prediction of STAT3/CDK2/4/6 as druggable candidates for NSC765690 and NSC765599. The present study, therefore, provides useful molecular docking-based evidence of alternative small molecules that could target CDK4/6 as well as CDK2 and STAT3. They thus offer a wider targeted therapy with less probability of suffering drug resistance

The concept of drug-likeness gives useful guidelines for identifying potential drug candidates during the early stage of drug discovery and development [59]. Estimation of rodent acute toxicity (LD_50_), an adverse effect that follows a single dose exposure to a substance, is an important task in drug design and risk assessment of chemicals [60,61,62]. The LD_50_ values predicted for oral, intravenous, intraperitoneal, and subcutaneous administration indicated that both NSC765690 and NSC765599 were non-toxic and will be well-tolerated by the experimental animals. In addition, NSC765690 and NSC765599 met the criteria of a good drug candidate (Table 7), suggesting that the compounds were drug-like molecules and thus possessed the potential to be considered oral drug candidates. Collectively, findings from this study have opened the door for new research direction for NSC765690 and NSC765599 as a novel and potent anti-cancer agent. We found these compounds worthy of further investigation, and both in vitro and in vivo studies in tumor-bearing mice are currently ongoing in our lab to assess the full therapeutic efficacies of these compounds.

## 5. Conclusions

In conclusion, this study demonstrated the anti-proliferative effect of NSC765690 and NSC765599 against the 60 panels of NCI human cancer cell lines, and dose-dependent cytotoxic preference for NSCLC, melanoma, renal, and breast cancer cell lines. STAT3/CDK2/4/6 signatures appear to be potential druggable candidates for the small molecules and interacted with high binding affinity, indicating the potential of NSC765690 and NSC765599 to dysregulate expressions of STAT3/CDK2/4/6 signature and, consequently, compromised cell survival. Further in-vitro and in-vivo confirmation studies are ongoing in our lab.

## Figures and Tables

**Table 1:** Anti-proliferative and cytotoxic activities of NSC765690 and NSC765599 against panels of NCI60 human cancer cell lines.

Cancer Type	Panel/Cell Line	GI_50_ (μM)		TGI (μM)		LC_50_ (μM)	
		NSC765690	NSC765599	NSC765690	NSC765599	NSC765690	NSC765599
Leukemia	CCRF-CEM	0.53	0.703	9.73	5.6	>100	>100
	HL-60(TB)	0.51	0.586	2.09	2.68	>100	>100
	K-562	0.37	0.628	10.2	19.1	>100	>100
	MOLT-4	0.46	0.928	2.05	3.39	>100	>100
	RPMI-8226	0.23	0.248	0.74	0.951	>100	>100
	SR	1.91	1.95	>100	8.27	>100	>100
NSCLC	A549/ATCC	0.44	1.05	7.96	10.6	>100	99.4
	HOP-62	1.61	2.51	6.78	7.48	>100	37
	HOP-92	0.14	0.219	0.48	0.655	>100	53.7
	NCI-H226	1.07	1.73	11.2	12	>100	>100
	NCI-H23	0.29	0.395	0.88	2.54	>100	94.2
	NCI-H322M	0.68	1.3	6.7	6.13	>100	>100
	NCI-H460	0.45	0.569	8.78	10.7	>100	>100
	NCI-H522	0.28		0.77		19.4	
Colon Cancer	COLO 205	3.23	4.48	11.2	15.6	35.8	44.4
	HCC-2998	2.16	2.42	6.31	5.83	30.6	21.2
	HCT-116	0.55	1.05	3.1	2.84	93.1	7.7
	HCT-15	0.48	1.22	4.05	11.4	45.9	>100
	HT29	3.63	3.43	10.6	9.93	36.5	36.6
	KM12	0.7	1.99	10.5	10.1	>100	75.5
	SW-620	1.28	2.1	16.5	13.7	>100	>100
CNS Cancer	SF-268	0.71	2.17	>100	24.6	>100	>100
	SF-539	0.53	1.78	10.8	13.7	67.8	63.8
	SNB-19	0.41	0.529	>100	15.2	>100	>100
	SNB-75	1.39		9.75		>100	
	U251	0.42	0.633	7.23	4.97	82.9	29.7
Melanoma	LOX IMVI	0.5	0.64	3.28	3.36	26.3	21
	MALME-3M	0.4	1.02	2.26	3.48	>100	>100
	M14	0.68	1.38	7.16	5.37	>100	93.7
	MDA-MB-435	0.63	1.31	6.04	4.5	>100	42.7
	SK-MEL-2	0.28	0.443	0.73	1.69	5.51	8.39
	SK-MEL-28	0.68	0.865	7.05	9.28	>100	>100
	SK-MEL-5	0.26	0.345	0.88	1.26	4.61	4.39
	UACC-257	0.29	0.815	1.59	4.84	>100	>100
	UACC-62	0.28	0.319	1.14	1.22	5.59	7.21
Ovarian Cancer	IGROV1	0.55	0.909	24.3	10.7	>100	>100
	OVCAR-3	0.44	0.674	33	15.3	>100	>100
	OVCAR-4	0.86	0.841	>100	18.5	>100	>100
	OVCAR-5	4.04	5.55	>100	>100	>100	>100
	OVCAR-8	0.51	1.64	6.01	10.1	>100	>100
	NCI/ADR-RES	0.42	0.741	10.4	9.96	>100	>100
	SK-OV-3	1.29	2.68	7.3	11.8	>100	>100
Renal cancer	786-0	2.72	2.58	9.07	7.45	>100	39.7
	A498	0.24	0.263	1.52	1.93	>100	>100
	ACHN	0.82	1.73	15.5	14.4	>100	>100
	CAKI-1	0.65	0.637	>100	4.07	>100	>100
	RXF 393	1.47	2.48	5.93	6.72	>100	35.2
	SN12C	0.37	0.384	12.7	4.73	>100	>100
	TK-10	0.45	1.46	4.01	6.87	>100	82.7
	UO-31	0.41	1.14	7.18	6.95	37.4	32.4
Prostate Cancer	PC-3	0.38	1.3	3.04	5.43	>100	42.8
	DU-145	1.28	2.16	>100	23.4	>100	>100
Breast Cancer	MCF7	0.42	0.471	9.11	13.4	>100	>100
	MDA-MB231/ATCC	1.06	4.76	4.08	50.5	>100	0
	HS 578T	0.65	1.31	28.7	8.65	>100	>100
	BT-549	0.45	1.11	2.59	5.5	43.6	48.8
	T-47D	0.29	0.449	2.74	7.49	>100	>100
	MDA-MB-468	0.3	0.425	3.17	4.42	>100	>100

The GI_50_ = 50% growth inhibition, TGI = total growth inhibition, LC_50_ = 50% loss of cells.

**Table 2:** NCI synthetic compounds and standard anticancer agent sharing similar anti-cancer fingerprints and mechanistic correlation with NSC765599 and NSC765690.

		NCI-Synthetic Compounds				NCI-Standard Agents			
Drugs	Rank	P	CCLC	Target Descriptor	MW (g/mol)	P	CCLC	Target Descriptor	Mechanism of Action
NSC765690 Fingerprints	1	0.61	42	Antineoplastic-643812	419.3	0.74	55	Actinomycin D	Transcription inhibitor
	2	0.58	49	Combretastatin A-4	316.3	0.61	58	Mitramycin	Transcription inhibitor
	3	0.55	49	N-(3-chloro-2-methylphenyl)-2-hydroxy-3-nitrobenzamide	306.7	0.6	59	Thioguanine	Inhibit cell cycle transition
	4	0.51	46	2-Methyl-4-(phenylimino)naphth(2,3-d)oxazol-9-one	288.3	0.58	58	Cisplatin	Inhibit DNA replication
	5	0.51	46	Resibufogenin, Methacrylate De	452.6	0.58	49	Morpholino-ADR	Tubulin inhibitor
	6	0.5	43	3-Nitro-2′,4′-Salicyloxylidide	286.2	0.56	59	5-Azacytidine	Tubulin inhibior
	7	0.48	49	5,7-Dichloro-3-hydroxy-3-[2-(4-nitrophenyl)-2-oxoethyl]-1,3-dihydro-2H-indol-2-one	381.2	0.53	58	Topotecan	DNA damage inducer
	8	0.46	58	4-ipomeanol	168.1	0.48	59	Doxorubicin (Adriamycin)	DNA damage inducer
	9	0.46	48	3,3′-Diethyl-9-methylthiacarbocyanine iodide	506.5	0.39	59	5-Fluorouracil	Inhibitor of DNA replication.
	10	0.44	44	2,2-Dibutyl-3-(p-tolylsulfonyl)-1,3,2-thiazastannolidine	462.3	0.33	59	Abemaciclib	Inhibit cell cycle transition
NSC765599 Fingerprints	1	0.62	47	N-(3-chloro-2-methylphenyl)-2-hydroxy-3-nitrobenzamide	306.7	0.36	57	Trametinib	MEK Inhibitor
	2	0.57	44	Uvaretin	378.4	0.3	57	Erlotinib HCL	Growth factor receptor inhibitor
	3	0.54	44	Eunicin	334.4	0.29	55	Vandetanib	Growth factor receptor inhibitor
	4	0.54	44	Resibufogenin derivative	452.6	0.28	56	Topotecan	DNA damage inducer
	5	0.54	45	1H,3H-Thiazolo(3,4-a)benzimidazole, 1-(2-chloro-6-fluorophenyl)-	304.8	0.27	55	Ixabepilone	microtubule inhibitor
	6	0.53	44	Combretastatin A-4	316.3	0.23	57	Abemaciclib	Inhibitor of cell cycle transition
	7	0.52	44	Nagilactone C	362.4	0.25	56	Idelalisib	phosphoinositide 3-kinase
	8	0.51	56	dichloroallyl lawsone	283.1	0.23	57	Pazopanib Hydrochloride	Growth factor receptor inhibitor
	9	0.51	56	Merbarone	263.2	0.21	57	Doxorubicin (Adriamycin)	DNA damage inducer
	10	0.48	55	5-Bromo-1-[[4-methylidene-5-oxo-2-(4-phenylphenyl)oxolan-2-yl]methyl]pyrimidine-2,4-dione	453.3	0.15	57	Palbociclib	Inhibitor of cell cycle transition

P: Pearson’s correlation coefficient. CCLC: Common cell lines count. MW: molecular weight.

**Table 3:** Molecular targets correlated to NSC765599 and NSC765690 activity.

P	CCLC	P	CCLC	Target ID	Gene Card Code	Target Description
NSC765599		NSC765690				
0.30	53	0.29	55	CG2399	CCNB1	Cyclin B1
0.30	56	0.32	58	CG2465	CCND1	Cyclin D1
0.29	51	0.31	52	CG2440	RARB	Retinoic Acid Receptor Beta
0.28	54	0.13	55	CG2585	CDH1	Cadherin-1
0.28	48	0.17	50	CG2558	FGFR1	Fibroblast growth factor receptor 1
0.28	55	0.28	57	CG2369	RAF1	Raf-1 Proto-Oncogene
0.23	50	-	-	CG2405	E2F4	E2F Transcription Factor 4
0.22	52	-	-	CG2555	CDKN2A	cyclin-dependent kinase inhibitor 2A
0.21	56	0.15	58	CG2357	MYCN	N-myc proto-oncogene protein
0.22	50			CG2499	CDC25A	Activator of cyclin dependent kinase 2/4
0.21	56	0.11	58	CG2531	CDK4	Cyclin dependent kinase 4
0.21	56	-	-	CG2448	TCL1A	T-cell leukemia/lymphoma protein 1A
0.20	49	-	-	CG2269	BTK	Bruton Tyrosine Kinase
0.15	56	0.16	58	CG2311	PIK3CB	Phosphatidylinositol-4,5-bisphosphate 3-kinase
0.14	55	-	-	CG2466	CCND2	Cyclin D2
0.12	56	0.1	58	CG2327	CDK6	Cyclin dependent kinase 6
-	-	0.13	57	CG2467	CDC25B	Activator of cyclin dependent kinase CDC2
-	-	0.18	58	CG2468	CDKN1A	Cyclin Dependent Kinase Inhibitor 1A

P: Pearson’s correlation coefficient. CCLC: Common cell lines count. Pearson’s correlation coefficient ranges from high −1 (negative correlation) to +1 (high positive correlation). The higher the positive value, the more positive correlation between gene expression and NSC765599 and NSC765690 activities.

**Table 4:** SwissTarget prediction of potential protein targets for NSC765690 and NSC765599.

Gene Name	Common Name	Uniprot ID	ChEMBL ID	Target Class
NSC765690 Targets				
CDK9/cyclin T1	CDK9, CCNT1	P50750 O60563	CHEMBL2111389	Other cytosolic protein
Cyclin-dependent kinase 1	CDK1	P06493	CHEMBL308	Kinase
Cyclin-dependent kinase 1/cyclin B	CCNB3 CDK1 CCNB1 CCNB2	Q8WWL7 P06493 P14635 O95067	CHEMBL2094127	Other cytosolic protein
Cyclin-dependent kinase 2	CDK2	P24941	CHEMBL301	Kinase
Cyclin-dependent kinase 4/cyclin D1	CCND1 CDK4	P24385 P11802	CHEMBL1907601	Kinase
Epidermal growth factor receptor erbB1	EGFR	P00533	CHEMBL203	Kinase
Signal transducer and activator of transcription 3	STAT3	P40763	CHEMBL4026	Transcription factor
Fibroblast growth factor receptor 1	FGFR1	P11362	CHEMBL3650	Kinase
Insulin receptor	INSR	P06213	CHEMBL1981	Kinase
MAP kinase ERK2	MAPK1	P28482	CHEMBL4040	Kinase
PI3-kinase p110-gamma subunit	PIK3CG	P48736	CHEMBL3267	Enzyme
Platelet-derived growth factor receptor	PDGFRA PDGFRB	P16234 P09619	CHEMBL2095189	Kinase
Rho-associated protein kinase 1	ROCK1	Q13464	CHEMBL3231	Kinase
Serine/threonine-protein kinase 11/16/Chk1/MST2	STK11/3/16/CHEK1	Q15831	CHEMBL5606	Kinase
Tyrosine-protein kinase ABL/ITK/JAK1/JAK2	ABL1	P00519	CHEMBL1862	Kinase
NSC765599 Targets				
Cyclin-dependent kinase 5/CDK5 activator 1	CDK5R1 CDK5	Q15078 Q00535	CHEMBL1907600	Kinase
Epidermal growth factor receptor erbB1	EGFR	P00533	CHEMBL203	Kinase
Cyclin-dependent kinase 2	CDK2	P24941	CHEMBL301	Kinase
Cyclin-dependent kinase 4/cyclin D1	CCND1, CDK4	P24385, P11802	CHEMBL1907601	Kinase
Hepatocyte growth factor receptor	MET	P08581	CHEMBL3717	Kinase
Inhibitor of NF-kappa-B kinase (IKK)	CHUK	O15111	CHEMBL3476	Kinase
Insulin-like growth factor I receptor	IGF1R	P08069	CHEMBL1957	Kinase
MAP kinase p38 alpha	MAPK14	Q16539	CHEMBL260	Kinase
CDK6/cyclin D1	CCND1, CDK6	P24385, Q00534	CHEMBL2111455	Kinase
PI3-kinase p110-alpha/p85-alpha	PIK3CA, PIK3R1	P42336, P27986	CHEMBL2111367	Enzyme
Receptor protein-tyrosine kinase erbB-2	ERBB2	P04626	CHEMBL1824	Kinase
Receptor protein-tyrosine kinase erbB-4	ERBB4	Q15303	CHEMBL3009	Kinase
Rho-associated protein kinase 1	ROCK1/2	Q13464	CHEMBL3231	Kinase
Signal transducer and activator of transcription 3	STAT3	P40763	CHEMBL4026	Transcription factor
Serine/threonine-protein kinase RIPK2	RIPK2	O43353	CHEMBL5014	Kinase
Tankyrase−1/2	TNKS, TNKS2	O95271, Q9H2K2	CHEMBL6164, 6154	Enzyme
Tyrosine-protein kinase JAK1	JAK1	P23458	CHEMBL2835	Kinase
Tyrosine-protein kinase SRC	SRC	P12931	CHEMBL267	Kinase
Vascular endothelial growth factor receptor 1/2	FLT1, KDR	P17948, P35968	CHEMBL1868, 279	Kinase

Targets were predicted using Swiss Target Prediction, which operates on the principle of ‘similarity’.

**Table 5:** Prediction of Biological Activity Spectra (PASS) of NSC765690 and NSC765699 Targets.

NSC765690 PASS Predicted Targets			NSC765699 PASS Predicted Targets		
Pa	Pi	Activity	Pa	Pi	Activity
0.446	0.034	CDK6/cyclin D1 inhibitor	0.505	0.012	Transcription factor inhibitor
0.430	0.037	Transcription factor STAT inhibitor	0.430	0.037	Transcription factor STAT inhibitor
0.266	0.085	Transcription factor STAT3 inhibitor	0.391	0.020	CDK6 inhibitor
0.167	0.038	CDK9/cyclin T1 inhibitor	0.255	0.072	Transcription factor STAT3 inhibitor
0.151	0.046	CDK2/cyclin A inhibitor	0.162	0.029	CDK2/cyclin A inhibitor
0.114	0.021	Transcription factor STAT6 inhibitor	0.114	0.021	Transcription factor STAT6 inhibitor
0.101	0.094	CDK1/cyclin B inhibitor	0.020	0.011	CDK4/cyclin D3 inhibitor
0.019	0.012	CDK4/cyclin D3 inhibitor	0.046	0.007	CDK5 inhibitor

Key: Pa > Pi, Pa: probability to be active, Pi: probability to be inactive.

**Table 6:** Comparative docking profile of NSC765690, NSC765599 and standard STAT3/CDK2/4/6 inhibitors.

	**Cyclin Dependent Kinase 2**					
**Docking Parameters**	**NSC765599_CDK2 Complex**		**NSC765690_CDK2 Complex**		**Palbociclib_CDK2 Complex**	
ΔG = (Kcal/mol)	−11		−11.6		−9.1	
Type of Interactions	*n*-Bond	Interacting AA (Distance (Ă))	*n*-Bond	Interacting AA (Distance (Ă))	*n*-Bond	Interacting AA (Distance (Ă))
Conventional H-bond	4	LYS129 (2.67), LYS33 (2.73), GLN131 (2.72) ASP86 (3.36),	3	LYS129 (2.75), LYS89 (2.15) LYS33 (2.14)	2	ARG214(2.16), ARG217 (2.65)
C-H bond					2	VAL251(3.70) LYS250 (3.70)
Halogen bond	2	HIS84 (3.69),GLN85 (3.36)				
Pi-cation	1	LYS89 (3.11)				
Pi-anion						
Pi-alkyl			2	LEU134, ILE10	2	PRO204, ARG200
Pi-pi stack						
Amide-pi stack	1	GLY13 (3.34)				
Van der waal forces	10	LEU83, PHE82, ALA144, VAL18, GLU12, VAL164, THR158, ALA31, THR14, ASN132,	14	PHE82, LEU83, ASP145, ALA144, ASN132, GLN131, THR14, THR158, GLU12, GLY13, GLY11, ASP86, GLN85, HIS84	5	GLN246, THR218, LEU202, THR198, LYS250
	**Cyclin Dependent Kinase 4**					
	**NSC765599_CDK4 Complex**		**NSC765690_CDK4 Complex**		**Palbociclib_CDK4 Complex**	
ΔG = (Kcal/mol)	−7.5		−8.5		−8.3	
Type of Interactions	*n*-Bond	Interacting AA (Distance (Ă))	*n*-Bond	Interacting AA (Distance (Ă))	*n*-Bond	Interacting AA (Distance (Ă))
Conventional H-bond	2	ARG172 (2.07) GLU66 (2.06)	4	ARG67(1.84), ALA162 (2.62) LYS179 (2.90) HIS158(2.87)	4	ARG168(3.23), SER57(2.49)
Halogen bond			2	ASP159 (2.90), HIS158(3.62)		
C-H bond					1	GLY51 (3.46)
Pi-cation			1	ARG67(3.89)		
Pi-anion	1	ASP173 (4.48)	1	ASP159 (3.78)		
Pi-alkyl	1	MET59	1	ALA153, ALA162	4	TYR172, PRO55, PRO45, ILE56
Van der waal forces	3	LEU60, LEU99, TRP63	10	LEU64, ARG60, LEU113, ALA63, LEU31, PHE163, PHE160, LEU161, GLU76, ARG114	11	SER171, GLY48, GLY53, GLY52, GLY50, THR120, ILE121, LYS112, LEU108, LEU54, THR58
	**Cyclin Dependent Kinase 6**					
	**NSC765599_CDK6 Complex**		**NSC765690_CDK6 Complex**		**Palbociclib_CDK6 Complex**	
ΔG = (Kcal/mol)	−9.0		−9.6		−8.1	
Type of Interactions	*n*-Bond	Interacting AA (Distance (Ă))	*n*-Bond	Interacting AA (Distance (Ă))	*n*-Bond	Interacting AA (Distance (Ă))
Conventional H-bond	1	GLU69 (2.22)	2	HIS139 (2.29) SER138 (2.31)	2	ARG66 (2.50) GLU69 (3.17)
C-H bond			1	PHE135(3.69)	1	PRO35(3.71)
Halogen bond	2	HIS73 (2.88) VAL76 (2.99)	1	ASN26 (3.32)		
Pi-cation	1	ARG66 (3.40)				
Pi-anion	1	GLU69 (4.90)	1	ASP134(3.99)	1	ASP146(3.59)
Pi-sigma					1	LEU34
Pi-alkyl			1	ARG131	4	ALA149, LEU33, ARG82, CYS85
Pi-pi stack	1	GLU72, ARG78	1	TYR292	1	PHE37292
Pi-pi T-shape			1	PHE135		
Van der waal forces	7	Lys160, Val77, Leu79, Phe80, Asp81, Val82, Lys144	5	Phe71, Ser296, Leu295, Phe127, His73	5	Lys36, Thr38, Thr70, Glu148, His67
	**Signal Transducer and Activator of Transcription 3**					
	**NSC765599_STAT3 Complex**		**NSC765690_STAT3 Complex**		**SH-4-54_STAT3 Complex**	
ΔG = (Kcal/mol)	−8.0		−8.3		−7.3	
Type of Interaction	*n*-Bond	Interacting AA (Distance (Ă))	*n*-Bond	Interacting AA (Distance (Ă))	*n*-Bond	Interacting AA (Distance (Ă))
Conventional H-bond	2	ARG107(2.73) TRP110 (3.79)			1	SER113 (2.57)
C-H bond			1	ARG107 (3.23)	2	TRP110 (3.69) ALA106 (3.47)
Halogen	1	GLN3 (3.52)	1	ALA106 (3.67)	3	ARG107(3.65), ARG103 (3.05), ALA44 (3.26)
Pi-pi stacked	1	TRP110				
Pi-sigma			1	ALA44		
Pi-sulfur					1	TRP110 (4.49)
Pi-alkyl	3	LEU109, ALA106, ALA44	3	LEU109, ARG103, ALA44	1	ALA106
Pi-pi T-shaped			1	TRP110		
Van der waal forces	5	GLU39, GLN41, ASP42, TYR45, TRP43	4	TRP43, GLU39, GLN3, SER113	4	GLU39, GLN3, GLN117, ALA47

Interacting AA: Interacting Amino acids.

**Table 7:** Drug likeness, ADME and safety/toxicity profile of NSC765690 and NSC765599.

Properties	NSC765690	NSC765599	Reference Value
Formula	C_21_H_10_F_2_N_2_O_3_	C_20_H_12_F_2_N_2_O_2_	-
Molecular weight	376.31 g/mol	350.32 g/mol	150–500 g/mol
Num. rotatable bonds	2	4	0–9
Num. H-bond acceptors	6	5	0–10
Num. H-bond donors	0	2	0–5
Molar Refractivity	98.15	92.75	
TPSA	76.00 Å²	73.12 Å²	20–130 Å²
Fraction Csp3	0.00	0.00	0.25~<1
Log P_o/w_ (XLOGP3)	4.31	5.47	−0.7~5
Consensus Log P_o/w_	4.11	4.29	
Log S (ESOL)	0.34	−5.71	0–6
Lipinski, Ghose, Veber and Egan’s rule	Yes; 0 violation	Yes; 0 violation	
Bioavailability Score	0.56	0.55	>0.1 (10%)
Synthetic accessibility	3.33	2.37	1 (very easy) to 10 (very difficult).
	Acute toxicity		
LD_50_ for Intraperitoneal (mg/kg)	446.100 (OECD:4)	593.200 (OECD:5)	
LD_50_ for Intravenous (mg/kg)	127.200 (OECD:4)	251.200 (OECD:4)	
LD_50_ for Oral (mg/kg)	494.000 (OECD:4)	836.900 (OECD:4)	
LD_50_ for Subcutaneous (mg/kg)	398.300 (OECD:4)	740.900 (OECD:4)	
	Environmental toxicity		
Bioaccumulation factor Log10 (BCF)	1.204	1.210	
Daphnia magna LC_50_Log10 (mol/L)	7.294	6.713	
Fathead Minnow LC_50_Log10 (mmol/L)	−3.088	−3.099	
Tetrahymena pyriformis IGC_50_Log10 (mol/L)	2.016	2.033	

## Data Availability

The datasets generated and/or analyzed in this study are available on reasonable request.

## Supplementary Materials

The following are available online at https://www.mdpi.com/2227-9059/9/1/92/s1. Figure S1: Pie chart showing the repartition of protein classes of potential druggable candidates for NSC765690 and NSC765599. Figures S2–S5: Docking profiles of NSC765690, NSC765599, palbociclib with CDK2/4/6 and STAT3. Figure S6: BOILED-Egg model of brain or intestinal estimated permeation of NSC765599 and NSC765690. Figure S7: Bioavailability Radar showing suitable physicochemical spaces of the oral bioavailability of NSC765690 and NSC765599.
