# Supplementary material for: Pharmacoinformatics and Preclinical Studies of NSC765690 and NSC765599, Potential STAT3/CDK2/4/6 Inhibitors with Antitumor Activities against NCI60 Human Tumor Cell Lines
Source: Biomedicines. 2021 Jan 19;9(1):92. doi: 10.3390/biomedicines9010092 (PMC7832910; doi:10.3390/biomedicines9010092)
Supplement: Supplementary file 1 [file biomedicines-09-00092-s001.pdf]

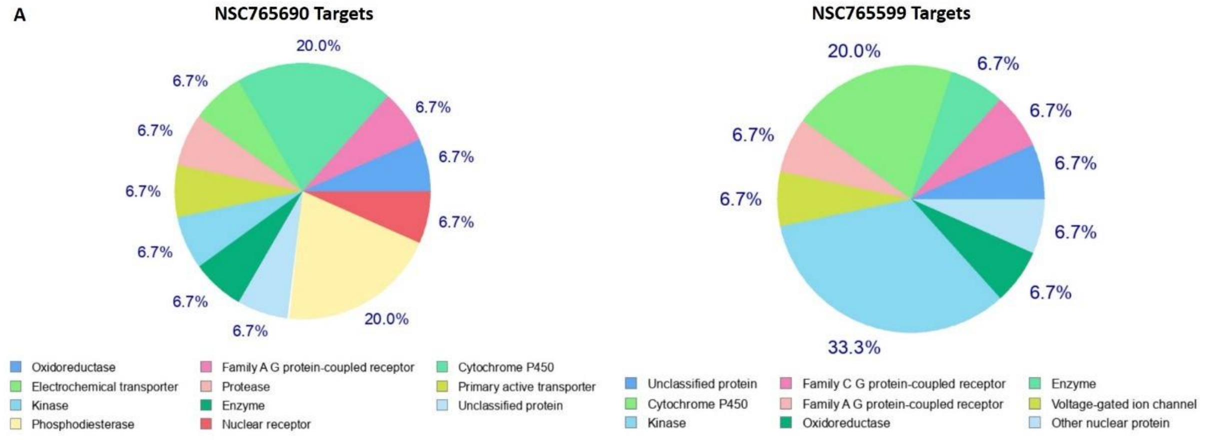

B

NSC765690 Targets	Common name	Uniprot ID	ChEMBL ID	Target Class
**CDK9/cyclin T1**	CDK9 CCNT1	P50750 O60563	CHEMBL 2111389	Other cytosolic protein
**Cyclin-dependent kinase 1**	CDK1	P06493	CHEMBL308	Kinase
**Cyclin-dependent kinase 1/cyclin B**	CCNB3 CDK1 CCNB1 CCNB2	Q8WWL7 P06493 P14635 O95067	CHEMBL 2094127	Other cytosolic protein
**Cyclin-dependent kinase 2**	CDK2	P24941	CHEMBL301	Kinase
**Cyclin-dependent kinase 4/cyclin D1**	CCND1 CDK4	P24385 P11802	CHEMBL 1907601	Kinase
**Epidermal growth factor receptor erbB1**	EGFR	P00533	CHEMBL203	Kinase
**Signal transducer and activator of transcription 3**	STAT3	P40763	CHEMBL4026	Transcription factor
**Fibroblast growth factor receptor 1**	FGFR1	P11362	CHEMBL3650	Kinase
**Insulin receptor**	INSR	P06213	CHEMBL1981	Kinase
**MAP kinase ERK2**	MAPK1	P28482	CHEMBL4040	Kinase
**PI3-kinase p110-gamma subunit**	PIK3CG	P48736	CHEMBL3267	Enzyme
**Platelet-derived growth factor receptor**	PDGFRA PDGFRB	P16234 P09619	CHEMBL 2095189	Kinase
**Rho-associated protein kinase 1**	ROCK1	Q13464	CHEMBL3231	Kinase
**Serine/threonine-protein kinase 11/16/ Chk1/ MST2**	STK11/3/16/ CHEK1	Q15831	CHEMBL5606	Kinase
**Tyrosine-protein kinase ABL/ITK/JAK1/JAK2**	ABL1	P00519	CHEMBL1862	Kinase

NSC765599 Targets	Common name	Uniprot ID	ChEMBL ID	Target Class
**Cyclin-dependent kinase 5/CDK5 activator 1**	CDK5R1 CDK5	Q15078 Q00535	CHEMBL1907600	Kinase
**Epidermal growth factor receptor erbB1**	EGFR	P00533	CHEMBL203	Kinase
**Cyclin-dependent kinase 2**	CDK2	P24941	CHEMBL301	Kinase
**Cyclin-dependent kinase 4/cyclin D1**	CCND1 CDK4	P24385 P11802	CHEMBL 1907601	Kinase
**Hepatocyte growth factor receptor**	MET	P08581	CHEMBL3717	Kinase
**Inhibitor of NF-kappa-B kinase (IKK)**	CHUK	O15111	CHEMBL3476	Kinase
**Insulin-like growth factor I receptor**	IGF1R	P08069	CHEMBL1957	Kinase
**MAP kinase p38 alpha**	MAPK14	Q16539	CHEMBL260	Kinase
**CDK6/cyclin D1**	CCND1 CDK6	P24385 Q00534	CHEMBL2111455	Kinase
**PI3-kinase p110-alpha/p85-alpha**	PIK3CA PIK3R1	P42336 P27986	CHEMBL2111367	Enzyme
**Receptor protein-tyrosine kinase erbB-2**	ERBB2	P04626	CHEMBL1824	Kinase
**Receptor protein-tyrosine kinase erbB-4**	ERBB4	Q15303	CHEMBL3009	Kinase
**Rho-associated protein kinase 1**	ROCK1/2	Q13464	CHEMBL3231	Kinase
**Signal transducer and activator of transcription 3**	STAT3	P40763	CHEMBL4026	Transcription factor
**Serine/threonine-protein kinase RIPK2**	RIPK2	O43353	CHEMBL5014	Kinase
**Tankyrase-1**	TNKS	O95271	CHEMBL6164	Enzyme
**Tankyrase-2**	TNKS2	Q9H2K2	CHEMBL6154	Enzyme
**Tyrosine-protein kinase JAK1**	JAK1	P23458	CHEMBL2835	Kinase
**Tyrosine-protein kinase SRC**	SRC	P12931	CHEMBL267	Kinase
**Vascular endothelial growth factor receptor 1**	FLT1	P17948	CHEMBL1868	Kinase
**Vascular endothelial growth factor receptor 2**	KDR	P35968	CHEMBL279	Kinase

Supplementary figure 1: Pie chart showing the repartition of protein classes of potential druggable candidates for NSC765690 and NSC765599.

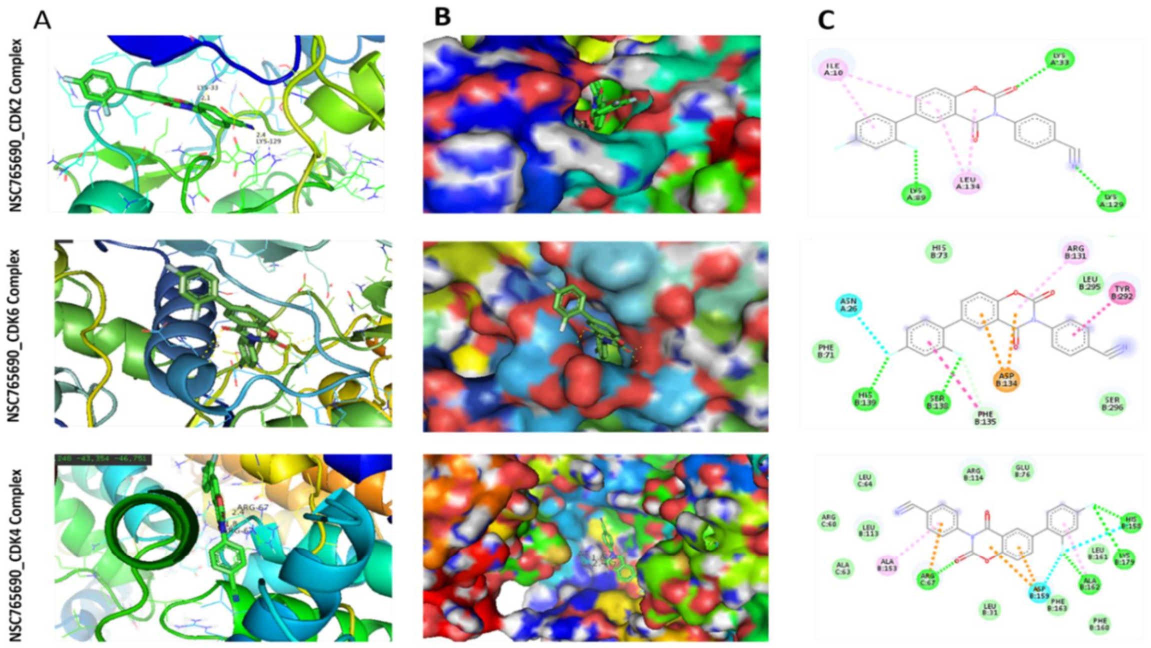

D

	Binding Energy (kcal/mol)	Interacting Amino acid	Binding Distance (Ă)	Interacting atom (Ligand-Receptor)
**CDK2**	-11.6	Lys 33	2.1	O – H
		LYS 129	2.4	N – H
		LYS 89	2.6	F – H
**CDK4**	-8.5	ARG 67	1.8	O – H
		ARG 67	2.4	O – H
		HIS158	2.1	F – H
		ALA162	3.2	F – H
		LYS179	432	
**CDK6**	-9.6	SER138	3.6	F – H
		HIS139	2.9	F – H

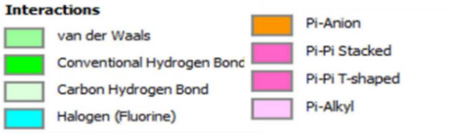

Supplementary figure 2: Docking profiles of NSC765690 with CDK2, CDK4, and CDK6. (A) The three-dimensional (3D) structures of ligand-receptor interactions of NSC765690 with CDK2, -4, and -6. (B) The surface representations of the active-site flap of the CDK2/4/6 complex with NSC765690 in the binding pocket.

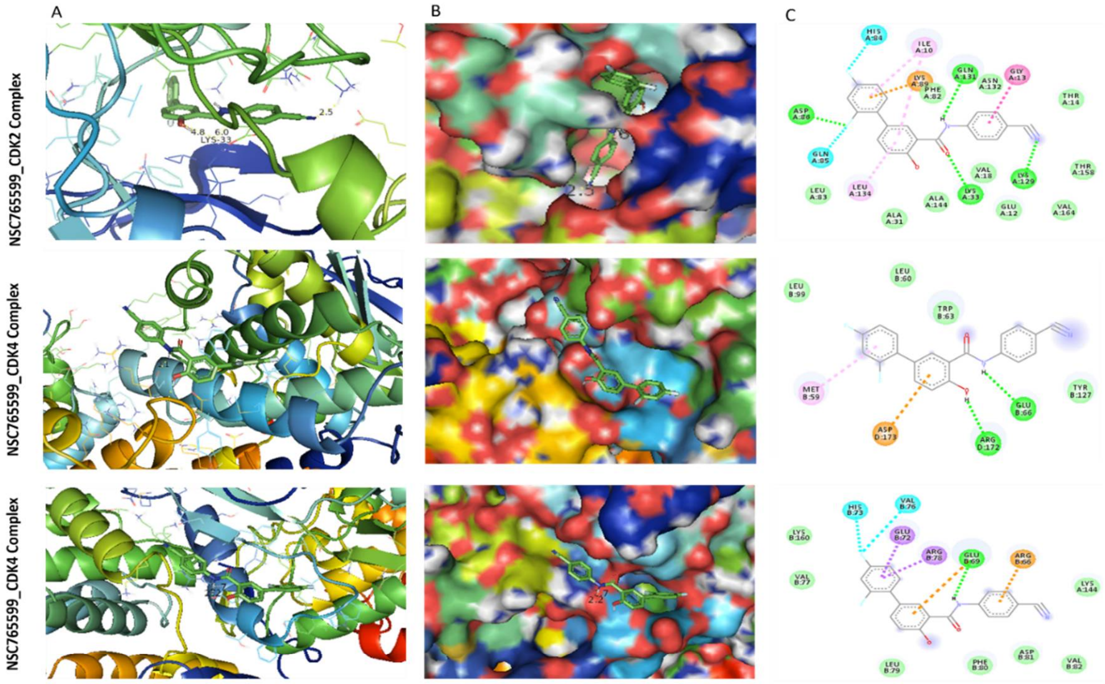

D

	Binding Affinity (Kcal/mol)	Interacting amino acid	Binding Distance (Å)	Interacting atom (Ligand-Receptor)
**CDK2**	-11.0	LYS33	2.2	O – H
		GLN 131	2.7	H – N
		LYS129	2.5	N – H
		ASP 86	3.7	H – O
**CDK4**	-7.5	GLU66	2.1	H – O
		ARG172	2.8	H – N
**CDK6**	-9.0	GLU69	2.1	H – O
		GLU69	2.7	H – O

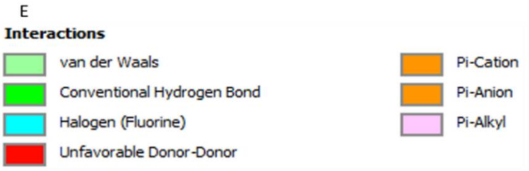

Supplementary figure 3: Docking profiles of NSC765599 with CDK2, CDK4, and CDK6. (A) The three-dimensional (3D) structures of ligand-receptor interactions of NSC765599 with CDK2, -4, and -6. (B) The surface representations of the active-site flap of the CDK2/4/6 complex with NSC765599 in the binding pocket.

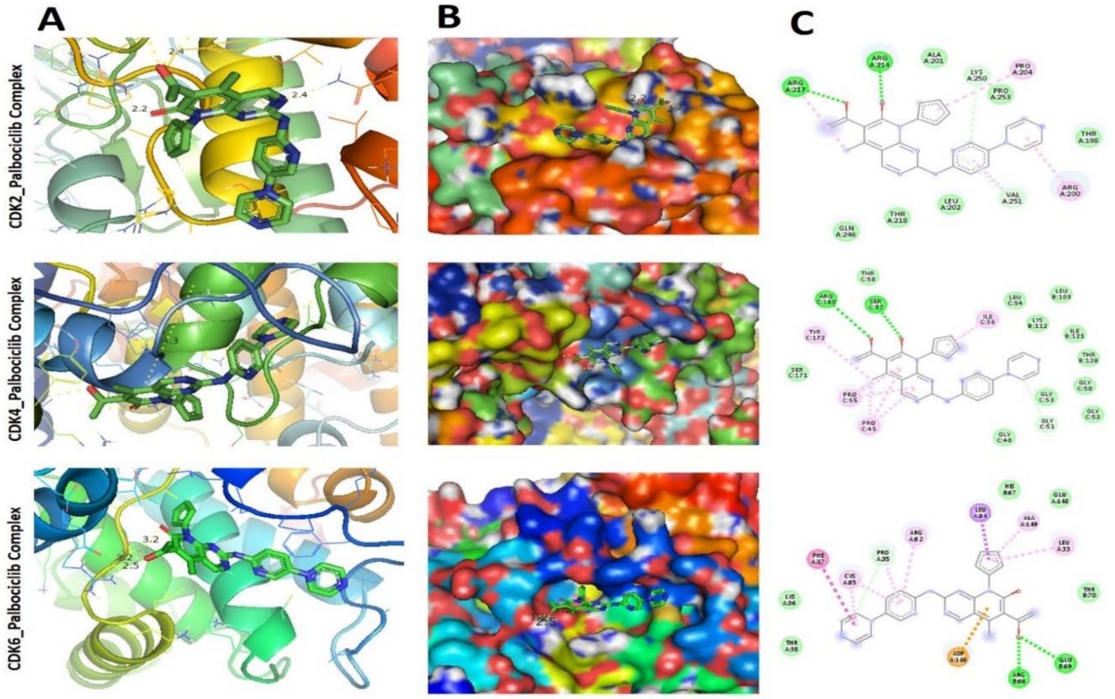

D

	Interacting amino acid	Binding Distance (Ǻ)	Interacting atom (Ligand -Receptor)	Binding Affinity (Kcal/mol)
**CDK2**	ARG 217	2.4	O – H	-9.1
	ARG 214	2.2	O – H	
	GLN 246	2.4	N – H	
**CDK4**	SER 57	3.8	O – H	-8.3
	ARG 168	4.3	O – H	
**CDK6**	GLU 69	3.2	O – H	-8.1
	ARG 66	2.5	O – H	

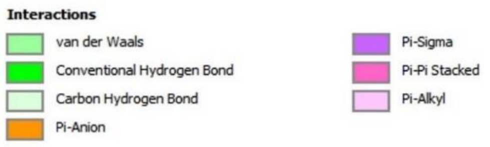

Supplementary figure 4: Docking profiles of palbociclib with CDK2, CDK4, and CDK6. (A) The three-dimensional (3D) structures of ligand-receptor interactions of palbociclib with CDK2, -4, and -6. (B) The surface representations of the active-site flap of the CDK2/4/6 complex with palbociclib in the binding pocket.

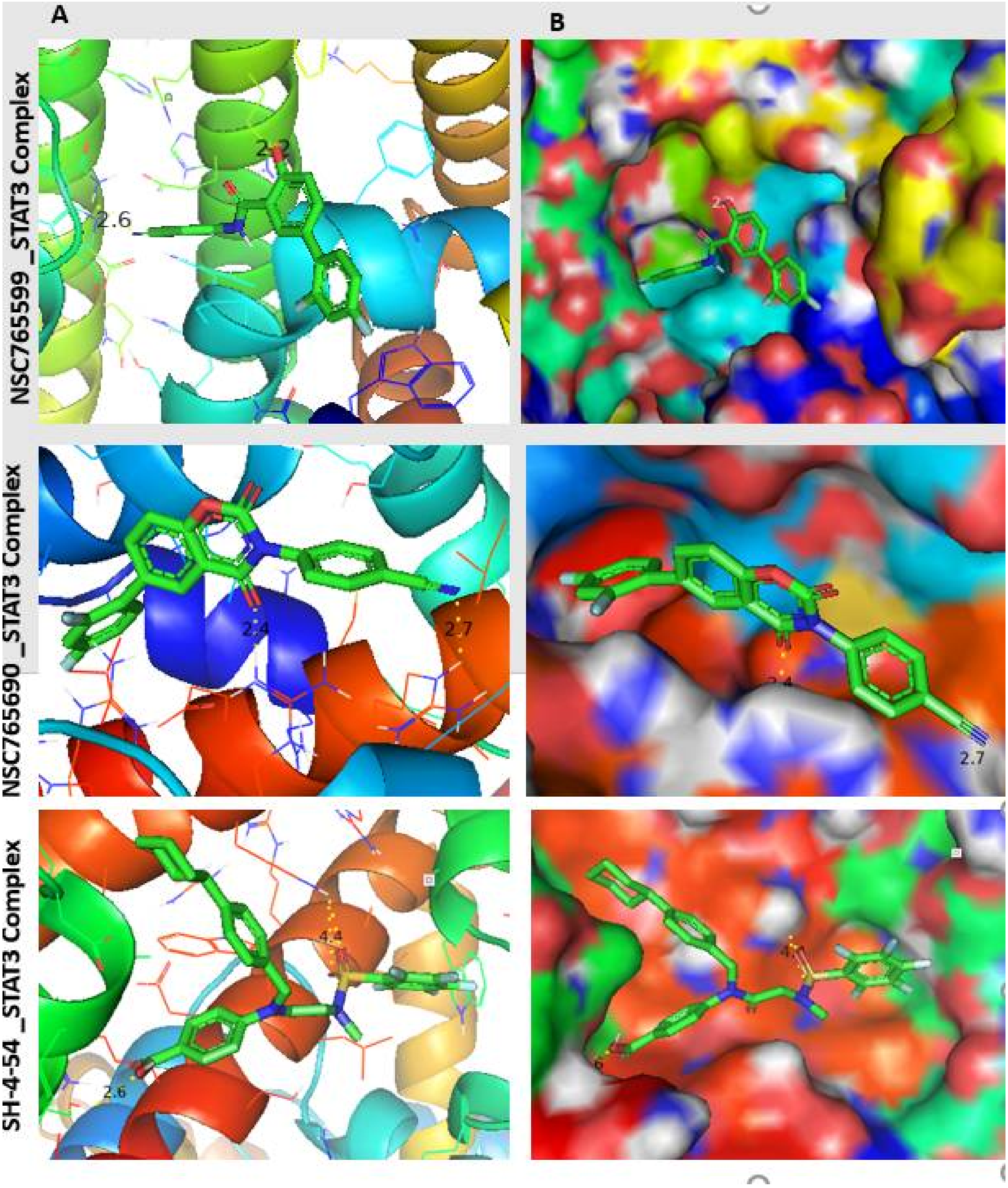

Supplementary figure 5: Comparative docking profiles of STAT3 with NSC765599, NSC765690, and SH-4-54 (a known STAT3 inhibitor). (A) The shows 3D structures of ligand-receptor interactions. (B) The show the surface representations of active-site flaps of the STAT3 complex with ligands in the binding pocket.

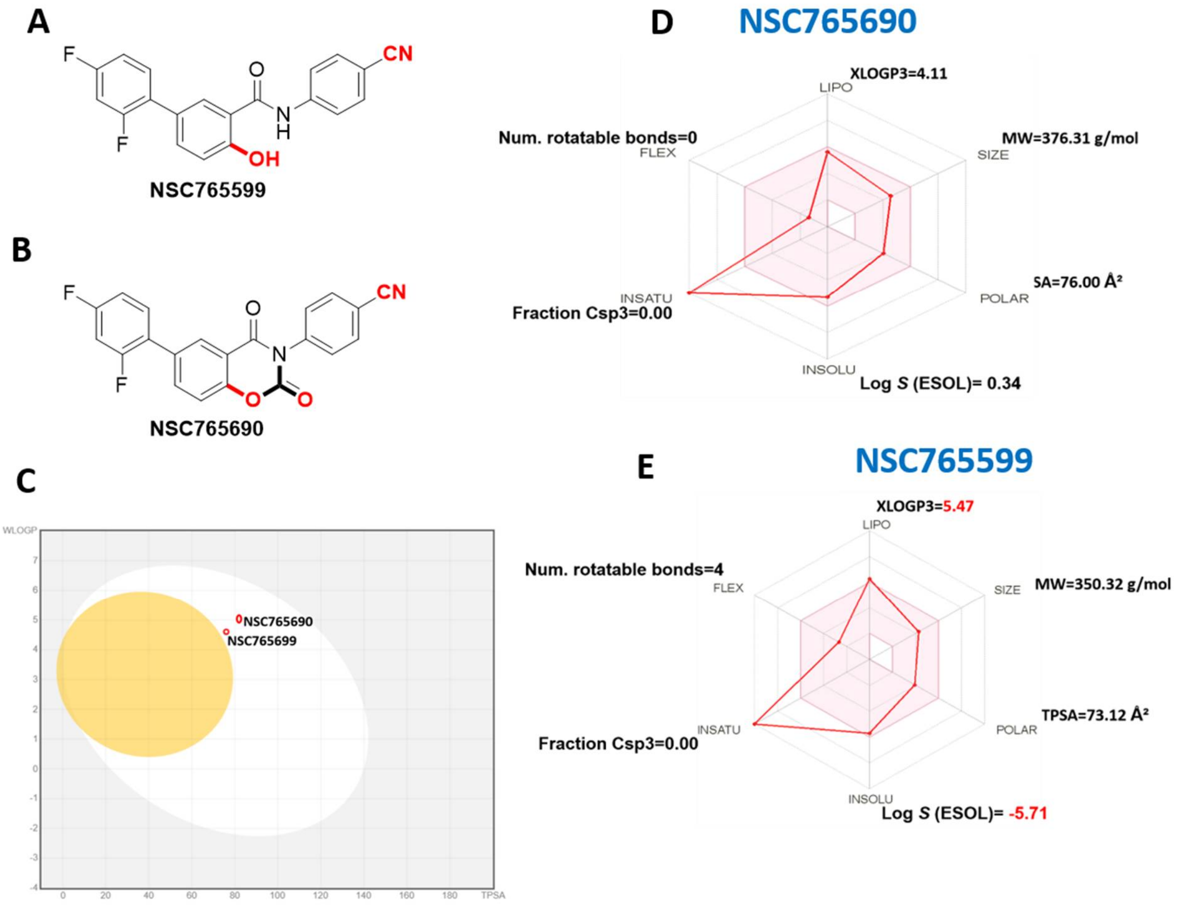

Supplementary figure 6: BOILED-Egg model of brain or intestinal estimated permeation of NSC765599 and NSC765690

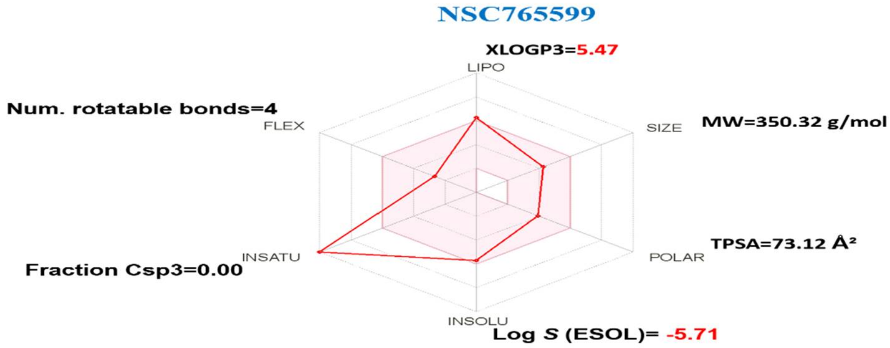

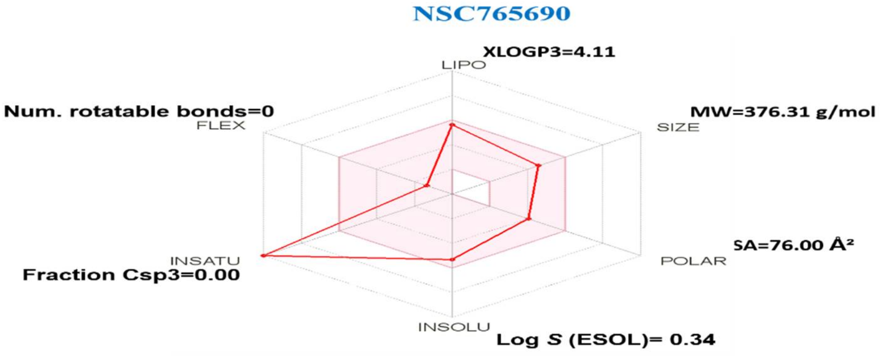

Supplementary Figure 7: Bioavailability Radar showing suitable physicochemical spaces of the oral bioavailability of NSC765690 and NSC765599. The pink area represents the optimal range for each property. LIPO, lipophilicity; POLAR, polarity; INSOLU, insolubility; INSATU, unsaturation; FLEX, flexibility; Fraction Csp3, fraction of carbons in sp3 hybridization.
